# Case Report: Congenital neurosyphilis presenting as post-hemorrhagic hydrocephalus in a preterm infant and a review of literature

**DOI:** 10.3389/fped.2025.1675980

**Published:** 2025-11-20

**Authors:** Domenico Umberto De Rose, Ludovica Martini, Maria Paola Ronchetti, Daniela Longo, Alessia Guarnera, Alessandra Santisi, Stefania Carrara, Alessandro De Benedictis, Venere Cortazzo, Carlo Federico Perno, Elvira Bonanno, Andrea Dotta, Cinzia Auriti

**Affiliations:** 1 Neonatal Intensive Care Unit, “Bambino Gesù” Children’s Hospital IRCCS, Rome, Italy; 2 Functional and Interventional Neuroradiology Unit, “Bambino Gesù” Children’s Hospital IRCCS, Rome, Italy; 3 Microbiology and Biobank Unit, National Institute for Infectious Diseases “Lazzaro Spallanzani”, IRCCS, Rome, Italy; 4 Neurosurgery Unit, “Bambino Gesù” Children’s Hospital IRCCS, Rome, Italy; 5 Microbiology and Diagnostic Immunology Unit, “Bambino Gesù” Children’s Hospital IRCCS, Rome, Italy; 6 Division of Neonatology and Neonatal Intensive Care Unit, “SS. Annunziata” Hospital, Cosenza, Italy; 7 Departmental Faculty of Medicine, Saint Camillus International University of Health and Medical Sciences, Rome, Italy

**Keywords:** congenital syphilis, ventriculomegaly, neurodevelopmental outcome, prematurity, preterm newborn, review, case report

## Abstract

**Background:**

Congenital syphilis (CS) remains a global public health concern, with rising incidence even in high-income countries. In Italy, a higher risk has been reported among primigravidae and younger mothers with late or missed prenatal screening. While neurological involvement in CS is well known, it is rarely reported in preterm infants, where it may be severe and atypical.

**Case presentation:**

A male preterm infant, delivered at 32 weeks via emergency cesarean due to abnormal fetal monitoring and breech position, was admitted to our NICU with intraventricular hemorrhage, midline shift, and hydrocephalus, requiring neurosurgery. He later developed a metaphyseal bone lesion; Staphylococcus aureus was found in blood cultures. Despite antibiotics, persistent thrombocytopenia and infectious symptoms led to further testing, revealing congenital syphilis with neurological involvement and osteomyelitis and osteochondritis of the distal ulna and radio from an undetected maternal treponemal infection during pregnancy. Penicillin therapy produced slow recovery, but the newborn developed epilepsy and spastic tetraplegia by 24 months. Genetic and metabolic tests were negative. Literature review rarely shows similar CS cases, especially in preterm infants.

**Conclusion:**

This case highlights the relevance of universal maternal syphilis screening and early neonatal evaluation. Maternal *Treponema pallidum* infection during pregnancy can result in preterm birth and may be associated with neurological complications, such as hemorrhages, seizures, and motor impairment, which can require multidisciplinary management and long-term follow-up.

## Background

In 2020, global congenital syphilis (CS) rates reached 425 cases per 100,000 live births, far exceeding the World Health Organization (WHO) target of 50 per 100,000 (1). High-income countries have also seen rising CS rates, with the US increasing from 11.6 to 102.5 cases per 100,000 between 2014 and 2022 (2). In Europe, Italian data show higher rates in younger mothers, first pregnancies, and late diagnoses, with paternal syphilis identified in only 36% of cases (3). The European Centers for Disease Control (ECDC) figures show CS cases in Italy dropped from 1.6 to 0.2 per 100,000 births between 2018 and 2020, though reporting may have been impacted by the pandemic. Most EU/EEA countries reported consistently low rates from 2012 to 2021, with six reporting no vertical transmission during this period (4). However, the estimates are not very accurate, and despite the existence of effective screening tools, cost-effective treatment options, and the incorporation of prevention programs into antenatal care, congenital syphilis continues to represent a significant global public health challenge.

We report the case of a preterm neonate presenting with early-onset post-hemorrhagic hydrocephalus, in whom congenital neurosyphilis was ultimately diagnosed. A brief review of the literature on central nervous system involvement in congenital syphilis is also provided. The diagnostic workup, guided by neuroimaging findings and laboratory results, led to targeted antibiotic therapy and multidisciplinary management.

## Case presentation

A male neonate was born at 32 weeks’ gestation via emergency cesarean section due to abnormal cardiotocographic (CTG) findings and breech presentation. The 32-year-old first-time mother had negative TORCH, hepatitis B, hepatitis C, and human immunodeficiency virus (HIV) infectious screening during pregnancy. Her vaginal swab at delivery was positive for an unspecified microorganism. The pregnancy was complicated by intrauterine growth restriction (IUGR) from week 31, oligohydramnios progressing to anhydramnios, and reduced fetal movements 48 h before birth. Two loops of the umbilical cord were noted around the neck at delivery. Birth measurements indicated small for gestational age: weight 1,360 g, length 39 cm, head circumference 28 cm (all below average per Italian INeS charts) (5). The infant required 17 days of mechanical ventilation, followed by CPAP and oxygen therapy. Cranial ultrasound revealed right temporo-parietal hemorrhage with midline shift and progressive left ventricular dilation (Levine index up to 2.7 cm). The initial bowel movement was delayed. Cholestatic jaundice was managed with ursodeoxycholic acid and minimal enteral feeding; *cytomegalovirus* DNA was negative. Empiric antibiotics (ampicillin, amikacin) were started, with ampicillin continued until the Neonatal Intensive Care Unit (NICU) transfer for surgery on day 22. Persistent thrombocytopenia was observed throughout the hospital stay.

**Figure 1:**
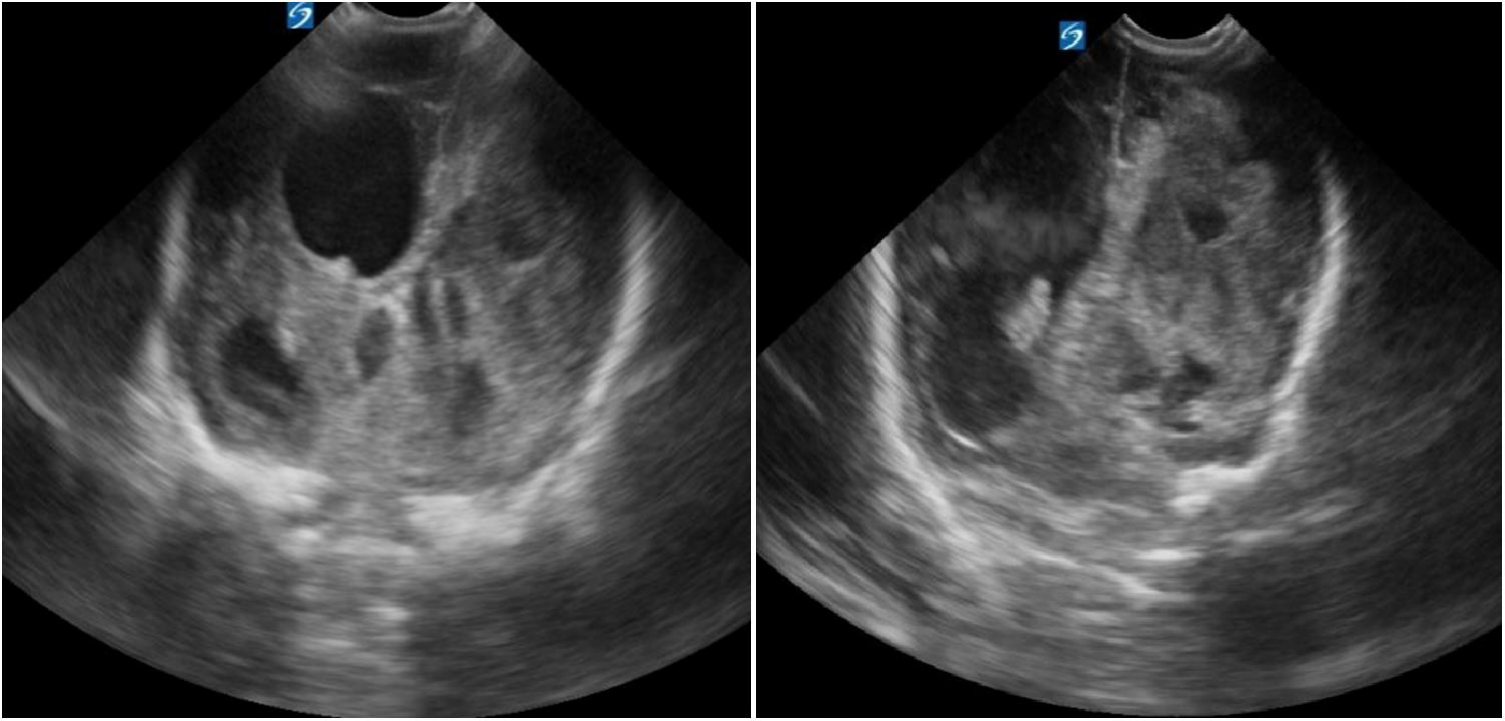
Cranial ultrasound upon admission to our NICU at 22 days of life.

Brain ultrasounds (US) at NICU admission revealed extensive right temporo-parietal hemorrhage with ipsilateral ventricular compression and leftward midline shift. Both lateral ventricles were enlarged (left: 29 mm; right: 13.2 mm), with blood clots present in the right lateral ventricle (Figure 1).

The day after admission, a small abscess-like lesion appeared on the left wrist, previously unobserved. x-ray showed significant osteostructural alterations of the distal ulna, with loss of distal margin definition and slight irregularity in the distal radial metaphysis. Ultrasound examination confirmed a mild irregularity of the ulnar metaphyseal contour. Despite no fever, osteomyelitis was suspected, so vancomycin and amikacin were started. Blood cultures identified *Staphylococcus aureus*.

Regarding hydrocephalus, a 6 mm Rickham ventricular catheter was inserted and connected to a left frontal subcutaneous reservoir, with regular CSF drainage procedures performed. Visual evoked potentials were within normal limits, and an electroencephalogram (EEG) showed no seizure activity.

After 20 days of antibiotic treatment, a moderate improvement was observed in the osteostructural changes at the distal metaphyseal end of the ulna. However, persistent neurological symptoms and the presence of VDRL positivity in breast milk, collected for donation from the mother, necessitated further investigations into infectious diseases for both the mother, father, and infant. Syphilis serology was performed on both parents, yielding the following results: in the mother, we noted RPR reactive at 1:16, reactive screening IgG/IgM, TPHA reactive at 1/2,500, Syphilis-specific IgG 23.42 IU/mL and IgM 3.99 IU/mL (normal ranges: 0–1); in the father, we found RPR reactive at 1:2, reactive screening IgG/IgM, TPHA reactive at 1/2,560, Syphilis-specific IgG 27.48 IU/mL and IgM 1.07 IU/mL (normal ranges: 0–1). Both tested negative for HIV-1 and HIV-2. These results led to a diagnosis of maternal syphilis during pregnancy, raising concerns about the risk of congenital syphilis in the infant. Parents received intramuscular Benzylpenicillin Benzathine 2,400 IU plus Betamethasone 4 mg weekly for 3 weeks.

The baby-boy was then subjected to laboratory screening for syphilis, including serological and molecular biology tests on blood and cerebrospinal fluid: CSF analysis via Western Blot showed positive IgM and IgG; VDRL was positive, and IgM by ELISA was negative. Polymerase Chain Reaction (PCR) test for Treponema pallidum on both cerebrospinal fluid (CSF) and blood from the patient returned negative results. Serological blood tests indicated positive IgG at 18.80 IU/mL (cut-off for positivity ≥1.0), RPR antibodies at 1:4 (cut-off for positivity >1:2), and TPHA antibodies at 1:320 (cut-off 1:80), due to a previously unrecognized and untreated maternal infection during pregnancy. Indeed, the mother was not previously tested for syphilis during pregnancy due to an inadvertent omission in the prenatal testing process.

The patient was subsequently treated with intravenous aqueous crystalline Penicillin G at a dosage of 50,000 units/kg for 10 days, resulting in the resolution of thrombocytopenia.

At the conclusion of the first treatment cycle, PCR on both CSF and blood was negative, IgM by ELISA was negative, and RPR had decreased to <1:2. However, Western Blot for both IgM and IgG remained positive in the CSF, and a second cycle of intravenous Penicillin G was administered for another 10 days. At a corrected age of approximately one month, a ventriculoperitoneal shunt was placed. By two months of corrected age, we observed a gradual decrease in IgM levels, and the patient was subsequently discharged. After the specific antibiotic treatment was described, CSF IgM levels turned negative, while both CSF and serum IgG levels remained persistently positive. PCR testing for Treponema pallidum in both serum and CSF consistently returned negative results throughout the follow-up period.

**Figure 2:**
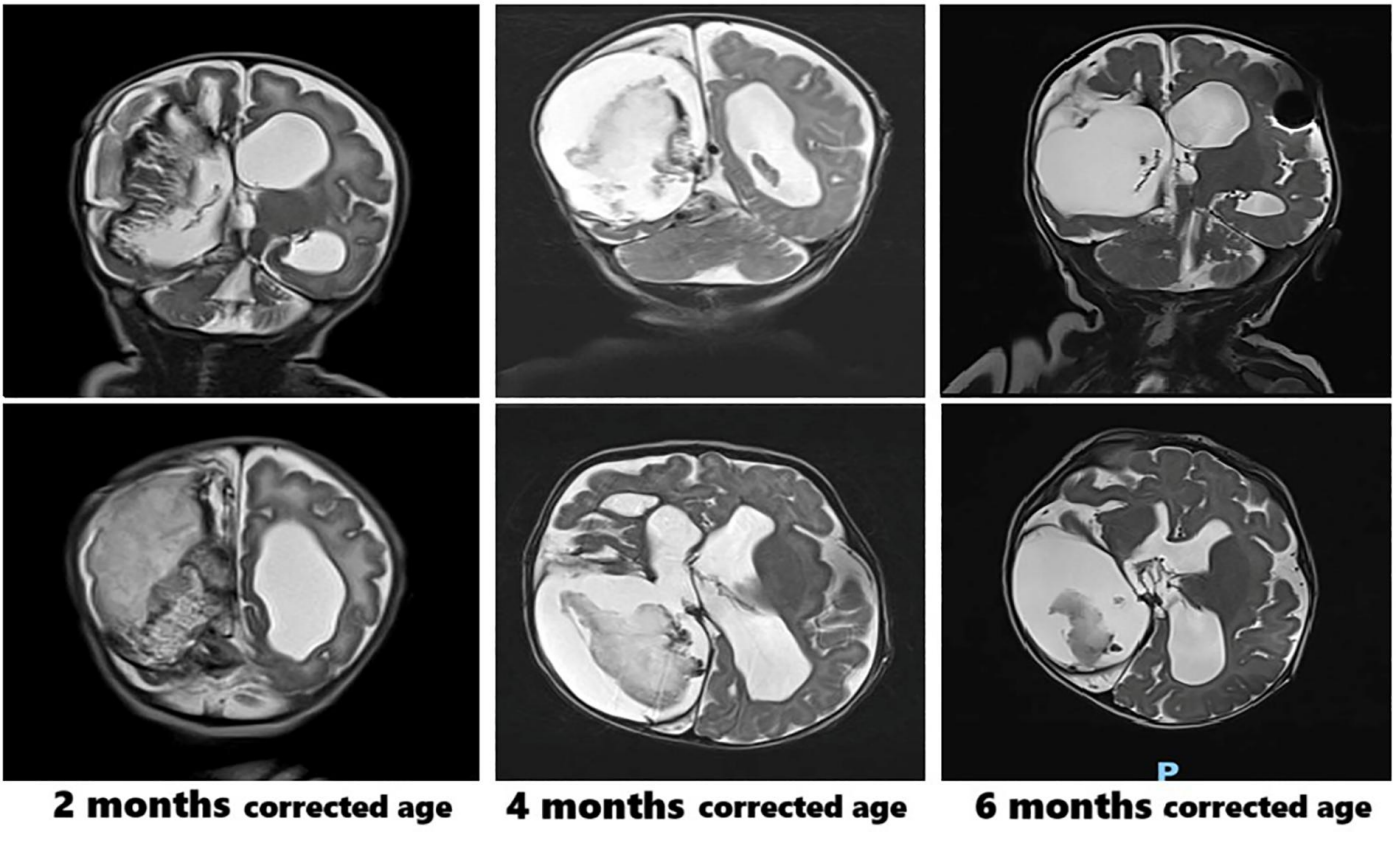
Brain MRI scans at 2, 4, and 6 months corrected age.

Clinically, the patient presented with macro-dolichocephaly, a prominent forehead, sunken eyes, and full cheeks. Karyotype analysis, array comparative genomic hybridization (CGH), and next-generation sequencing (NGS) for genes associated with syndromic cardiomyopathies (considering the observed biventricular cardiac hypertrophy, which resolved within one month following treatment) returned negative results. Serial brain MRI scans were performed during follow-up, as shown in Figure 2. The brain MRI at six months of corrected age showed a reduction in the size of the blood clot within the right endoventricular space. Other findings remained stable, including the enlargement of the supratentorial ventricular system, with the distal tip of the shunt catheter positioned in the right lateral ventricle.

At nine months of age, the infant began experiencing episodes resembling limb spasms that affected all four extremities. An EEG revealed slow activity accompanied by subcontinuous epileptiform abnormalities in the right frontal regions, prompting the initiation of intramuscular ACTH therapy. By eleven months, the EEG exhibited asymmetric cerebral activity, characterized by more disorganized patterns on the right side, with persistent slow and epileptiform abnormalities present in the right fronto-central regions during both wakefulness and sleep. Consequently, ACTH therapy was discontinued, and treatment with carbamazepine was initiated.

A neurodevelopmental evaluation at 24 months of corrected age indicated cerebral palsy (spastic tetraplegia), along with postural asymmetry resulting from right-sided plagiocephaly and increased overall muscle tone, particularly in the upper limbs. During the bilateral traction maneuver, the head remained tilted backward. In the prone position, the infant's upper limbs were mobilized slowly, requiring facilitation (via head rotation). The child occasionally managed to lift the head off the surface. An individualized postural support system was created for both indoor use and outdoor mobility.

## Methods

A MEDLINE (PubMed) search was conducted from 2000 to July 9th, 2025, using the keywords “congenital syphilis,” “central nervous system,” “neurosyphilis,” and “brain.” Only English-language papers with full case descriptions were included. We collected and summarized data on patient characteristics, neurological involvement, and procedures.

## Results

Table 1 summarizes published cases of neurological involvement in congenital syphilis during the neonatal period. Including our case, 16 cases were identified; 11 (68.8%) were preterm born. Neurological issues included cerebral hemorrhages, ventriculomegaly, Treponema pallidum DNA in cerebrospinal fluid, seizures, and hydrocephalus, along with systemic symptoms like hepatosplenomegaly, rash, and bone lesions. Outcomes ranged from normal development to neurodevelopmental delay, cerebral palsy, or death, regardless of maternal treatment or neonatal therapy (3, 6–17).

**Table 1:** Literature reports of neonatal congenital syphilis with neurological involvement compared to our case.

Author, year	Gestational age (weeks)	Birthweight (grams)	Timing of diagnosis and maternal treatment	Neurological findings	Other clinical findings	Neonatal treatment	Outcome
Filippi et al., 2004 (6)	33	1,150	Positive serological tests at 12 weeks of pregnancy, treated with erythromycin.	Hemorrhagic lesion in the right frontal lobe with enlargement of the right lateral ventricle's frontal horn, secondary to ongoing cavitation.	Erythemathous maculo-papular rash, hepatomegaly, chronic cholestatic liver disase.	No	No signs of cerebral palsy at 1 year of life.
Filippi et al., 2004 (6)	30	1,400	Anogenital ulcer with regional lymphadenopathy at 24 weeks, treated with benzathine benzylpenicillin at 26 weeks.	Left terminal vein rupture causing cerebral hemorrhage, bilateral intraventricular bleeding, and small bleeds in the basal ganglia and thalamus.	Bilateral pleural effusion, ascitic fluid at birth, cholestasis.	None	Death
Filippi et al., 2004 (6)	35	1,960	Positive maternal serology at 24 weeks; treated with benzathine benzylpenicillin.	Widened lateral ventricles with irregular borders, accompanied by expanding parenchymal cavities, disorganized background rhythm, and bilateral paroxysmal waveform abnormalities.	Not reported.	Intravenous penicillin G.	No reported long-term outcome
Tagarro et al., 2005 (7)	32	1,550	Maternal RPR negative in first trimester. Diagnosis of Congenital syphilis at day 10 of life.	Tremors, myoclonus, startles, mild ventriculomegaly, periventricular hyperechogenicity, subependymal hemorrhages, punctate calcifications (MRI)	Hydrops fetalis, anemia, thrombocytopenia, hepatosplenomegaly, osteochondritis, microalbuminuria.	Intravenous penicillin G.	At 9 months (corrected age), neurodevelopment was appropriate per Denver II, but the patient had microcephaly (z-score −3.69 SDS), tremors, hyperreflexia, and mild hypertonia; long-term outcome unknown.
Silva et al., 2012 (9)	Term	NA	Maternal syphilis seroconversion in 2nd trimester; treated with 3 intramuscular penicillin G doses; RPR negative before delivery.	Hypotonia, seizures, ventricular abnormalities on ultrasound and MRI, subdural hemorrhage, confirmed neurosyphilis by CSF PCR	No skin/mucosal signs; initial serology suggestive of passive maternal antibodies	Intravenous penicillin G.	At 12 months: global developmental delay, spastic diplegic cerebral palsy requiring rehabilitation, no further seizures.
Bembry et al., 2018 (10)	40	2,421 (small for gestational age)	RPR results were negative on three separate occasions.	Nonspecific bilateral diffuse subcortical edema without enhancement at brain MRI	Maculopapular desquamative rash, fever, enlarged liver and spleen, Hutchinson's triad (blunted upper incisors, interstitial keratitis, eighth nerve deafness), saddle nose, hard palate defect, rhagades, and diffuse periosteal reaction of long bones.	Intravenous penicillin G.	No information on the long-term outcome.
Greenall et al., 2020 (8)	29	1,340	Positive serological tests at 21 weeks.	Seizures that successfully resolved with phenobarbitone, ventriculitis	Respiratory distress syndrome, massive hepatomegaly, bony destructive changes at the proximal and distal ends of the femurs, proximal tibias, and proximal left humerus consistent with metaphysitis, thrombocytopenia, severe hypotension, hypoglycemia	Benzylpenicillin.	Discharge at 10 weeks, with no reported long-term outcome
Lee et al., 2020 (11)	32	1,460	Negative screening test 6 months before delivery, and no treatment for syphilis	Bilateral intraventricular hemorrhage (up to grade 2)	Desquamative rash, massive abdominal distension requiring ileostomy, hepatosplenomegaly, hemolytic anemia, leukocytosis, diffuse osteochondritis.	Intravenous penicillin G.	No information on the long-term outcome.
Iskandar et al., 2022 (13)	38	2,520	Maternal syphilis diagnosed in the 3rd trimester but untreated; neonatal diagnosis on admission	Decreased consciousness, seizures, CSF VDRL positive (neurosyphilis)	Respiratory distress, hepatosplenomegaly, jaundice, thrombocytopenia, elevated C-reactive protein, and liver enzymes (CS initially misdiagnosed as bacterial sepsis)	Intramuscular benzathine penicillin G for 3 weeks	Clinical improvement; no residual neurological symptoms at 30 days; jaundice gradually resolved; no information on the long-term outcome
Salomè et al., 2022 (3)	36	2,490	Diagnosis at birth; mother untreated	Seizures on EEG, CSF+: protein 167 mg/dl, WBC 113/mm³, NTT+	Hepatosplenomegaly, thrombocytopenia, coagulation disorder, anemia, periostitis and osteitis of tibia	Intravenous penicillin G.for 14 days	Normal neurological outcome; persistent radiological abnormalities without clinical signs
Serra et al., 2022 (15)	32	1,590	Positive TPHA and VDRL during the first trimester of pregnancy, but the mother did not undergo any treatment	Lateral ventricles dilation, diffuse periventricular hyperechogenicity with millimetric cavitated lesions, also within choroid plexuses and the germinative matrix	Gastrointestinal conditions included feeding difficulties, colon stenosis, and malabsorption, that resulted in postnatal growth restriction. Other associated findings were hypertrophic cardiomyopathy and hepatosplenomegaly.	Benzathine penicillin G.	Neurodevelopmental at 6 months within limits (mild delay due to an increased passive tone of the lower limbs), no information on the long-term outcome.
Tonni et al., 2022 (14)	NA	1,470	Negative maternal treponemal and non-treponemal tests	Bilateral intraventricular hemorrhage (grade III-IV), clinical signs of hypoxic-ischemic encephalopathy (HIE) with seizures	Pulmonary hemorrhage, anemia, cholestasis, systemic hypotension, and renal failure	Benzathine benzylpenicillin.	Death at 7 days of life.
Castelbranco-Silva et al., 2023 (12)	29	1,260	Positive maternal serological tests at birth	Post-hemorrhagic hydrocephalus, hypopituitarism, and diabetes insipidus	Petechial and purpuric rash, hepatosplenomegaly, and hydrops.	Intravenous penicillin G.	No reported long-term outcome
Newbery et al., 2023 (16)	37	3,325	Diagnosis at 23 weeks due to headache and visual/auditory symptoms; treated with 14 days intravenous penicillin G + 2 doses intramuscular benzathine penicillin G	Neurosyphilis was confirmed via cerebrospinal fluid analysis; no neurological abnormalities were identified in the fetus.	Calvaria osteitis (lytic skull lesions), right sensorineural hearing loss.	Single dose IM penicillin G to neonate; monitoring of neonatal RPR (1:4, lower than maternal 1:8).	Normal newborn clinical evaluation. Maternal lesions resolved by 4 months
Costa de Vasconcelos et al., 2024 (17)	39	3,475	Syphilis diagnosed in first trimester and treated; infant diagnosed with neurosyphilis postnatally (CSF VDRL 1:32, 64 cells/mm³)	Alterations in the cerebrospinal fluid test.	Hyperbilirubinemia requiring phototherapy.	Intravenous penicillin G.	Mild neurodevelopmental delay (expressive language and fine motor); preserved hearing; NIRS showed bilateral anterior cortical activation.
De Rose et al., 2025	32	1,360	Diagnosis at 3 weeks of life; maternal syphilis previously unrecognized and untreated during pregnancy.	Right temporo-parietal hemorrhage with midline shift and hydrocephalus, treated with ventricular reservoir and later ventriculoperitoneal shunt.	IUGR, oligo-anhydramnios, cholestatic jaundice, osteomyelitis (left ulna), thrombocytopenia, macrodolichocephaly, transient biventricular cardiac hypertrophy.	Intravenous penicillin G (2 courses).	Spastic tetraplegia, epilepsy, neurodevelopmental delay at 24 months.

## Discussion

In this manuscript, we compare the neurological involvement of a preterm newborn with congenital syphilis to cases described in the literature. The findings highlight the importance of universal maternal syphilis screening during pregnancy, as recommended by the World Health Organization, due to the availability of specific treatments. Mothers are advised to undergo serologic screening for syphilis during pregnancy. If the screening results are positive, treatment with Penicillin G is mandatory. Newborns should be examined at birth or before discharge, in accordance with the current recommendations.

In Italy, national guidelines mandate that mothers be tested for syphilis before pregnancy or at their first prenatal visit if not previously tested. In our patient's history, an inadvertent omission occurred in the prenatal testing process. While other routine prenatal examinations were performed, syphilis serology was not conducted prior to delivery or at the baby's discharge from the Neonatal Intensive Care Unit of the referral hospital. This underscores the critical need for adherence to infection prevention protocols during pregnancy, as well as to national and international guidelines. Our patient's history illustrates that, despite access to prenatal care, organizational factors can lead to missed prevention opportunities, highlighting the importance of strict compliance with current recommendations.

In the case of a neonatal infection, intravenous aqueous penicillin G for ten consecutive days is the treatment of choice. Our patient is distinguished from others by an exceptionally severe and multisystemic manifestation of congenital neurosyphilis, characterized by early-onset cerebral hemorrhage, post-hemorrhagic hydrocephalus necessitating neurosurgical management, ventricular reservoir and shunt placement included, and persistent neurological consequences such as spastic tetraplegia and epilepsy. Our initial clinical suspicion focused on post-hemorrhagic hydrocephalus secondary to prematurity. Subsequent findings revealed that previously undiagnosed and untreated maternal syphilis resulted in congenital infection in the newborn, as well as increased the risks of prematurity and neurological complications.

Although previous reports have described intracranial hemorrhages (6, 11, 14) in newborns affected by congenital syphilis, we have described the first case showing both anatomical brain damage and persistent neurological impairment at 24 months. Unlike Newbery et al. (16) or Costa de Vasconcelos et al. (17), where maternal infection was diagnosed and treated before birth, prenatal diagnosis was missed in our case, likely worsening neonatal outcomes. Our patient exhibited all systemic complications associated with congenital syphilis, including osteomyelitis, osteochondritis, cholestatic jaundice, intrauterine growth restriction, and transient biventricular cardiac hypertrophy. In our patient, karyotype analysis, array-CGH and NGS of genes associated with syndromic cardiomyopathies all returned negative results: no underlying genetic disorder was detected and this supports that the cardiac findings were most likely related to congenital syphilis. These clinical manifestations resulted in more severe presentation, compared to the milder cases documented by Iskandar et al. and Tagarro et al. (7, 13), where early diagnosis and treatment significantly improved prognosis.

Castelbranco-Silva et al., described a case of CS with post-hemorrhagic hydrocephalus, hypopituitarism, and diabetes insipidus, indicating how CS can lead to endocrine dysfunction, particularly hypopituitarism in infants (12). Previously, Daaboul et al. reported this finding in two infants with CS and persistent hypoglycemia who were found to have hypopituitarism. Affected infants with persistent hypoglycemia should receive a prompt evaluation of pituitary function (18).

Concerning cardiac findings of our patient, cardiac involvement in congenital syphilis is uncommon but has been reported in the literature. Documented findings include ventricular hypertrophy with ST-segment changes, aortic aneurysms, dilated cardiomyopathy, decreased ejection fraction, valvular disease with severe aortic regurgitation. These manifestations are generally associated with systemic inflammation and multisystem involvement, highlighting the importance of thorough cardiovascular evaluation in affected neonates (19).

Regarding the bone disease, it was diagnosed as acute osteomyelitis and attributed mainly to the *S. aureus* infection (as confirmed by blood culture). The lesion slowly improved after targeted antibiotic treatment. However, the patient exhibited also osteochondritis of the radius and ulna, characterized by a champagne-cup enlargement of the distal metaphysis of both the radius and ulna, which is indicative of a bone pathology likely resulting from congenital syphilis. Infants with CS can have skeletal system involvement in the form of osteochondritis or periostitis, with or without pseudoparalysis of Parrot (decreased range of movement due to painful periostitis) (20).

Furthermore, it is essential to repeat the non-treponemal serology test, even for women who tested negative during their initial visit, and in the case of positivity, start early antibiotic therapy, which is vital to prevent serious neonatal consequences. The infection was transmitted to the mother probably during the fifth month of her pregnancy, as per a retrospectively reconstructedtimeline.

In the last twenty years, this disease is re-emerging, partly due to migration from less developed areas, and disparities in healthcare access (driven by linguistic, cultural, and economic barriers) affect migrant populations and in the world, we are so far from the WHO target of fewer than 50 cases per 100,000 live births, established in 2007. In Italy, it is difficult to obtain precise estimates. A national survey in 2007 estimated the prevalence of syphilis seropositivity among pregnant women at 0.17%, with an incidence of congenital syphilis of 20 cases per 100,000 live births (21).

A further challenge is the diagnosis in preterm infants: early detection and treatment are critical. As we known, maternal infection in the first or early second trimester treated adequately with penicillin is associated with a lower risk of severe neonatal manifestations (6, 16, 17). Delayed diagnosis or incomplete treatment significantly increases the risk of vertical transmission and severe neonatal complications, of whom prematurity as the first of all (11, 15). Preterm neonates often present atypically, with milder or absent skin manifestations despite severe systemic or neurological disease. Maculopapular or desquamative rashes are common (6, 10, 11), as well as hepatosplenomegaly and cholestasis were frequently reported (6, 7). Thrombocytopenia, anemia, and coagulopathy can also occur in preterm infants (3, 7). Furthermore, hydrops fetalis and multi-organ compromise has been seen in severe untreated cases (7, 12).

Laboratory diagnosis can be also complicated by passive maternal antibodies, low birth weight, and immature immune responses (9).

Long-term monitoring is crucial, including neurodevelopmental assessments, hearing tests, and skeletal follow-up, given the already increased risk of delayed sequelae in these infants. Furthermore, infants exposed to syphilis prenatally, although if asymptomatic at birth, can present neurodevelopmental sequelae (22).

This report highlights the diagnostic complexity of congenital syphilis when classical signs are absent or delayed and underscores the importance of maintaining a high index of suspicion, especially in settings with incomplete or missing maternal serologic screening. We provided a detailed longitudinal description of neuroimaging findings, clinical course, and developmental outcomes over two years, contributing valuable insights into the possible evolution of congenital neurosyphilis in the preterm brain. Moreover, our patient shows the possible occurrence of delayed-onset seizures and spastic tetraplegia, which are rarely reported in association with CS, and links them to early hemorrhagic and ischemic brain injury. This experience sustains the need for universal syphilis screening during pregnancy, timely diagnosis, and multidisciplinary management. We must be aware of the possibility that neonates may have a poor outcome if congenital syphilis is not treated during pregnancy and after birth, especially in the case of preterm newborns.

Early diagnosis and timely treatment of congenital syphilis could improve neurodevelopmental outcomes and reduce the long-term healthcare burden on families and society by limiting the need for future medical, rehabilitative, and social support services (23).

## Conclusion

Congenital syphilis is associated with a high risk of severe multisystem complications in newborns, often with atypical presentations, especially in preterm infants. Neurological involvement in CS among preterm infants can be particularly severe, with reported cases of intraventricular hemorrhages, and ischemic brain lesions, underscoring the need for early neuroimaging and close neurological follow-up.

This highlights the need of prenatal screening of pregnant women, of retest them before delivery, of the timely therapy with Penicillin, in appropriate formulation and doses, in the case of infection and of the appropriate multidisciplinary follow-up for children at least up three years of life. In accordance with the recommendations from the U.S. CDC, we again emphasize that no neonate should be discharged from a neonatology unit without precise information on the maternal serological status for syphilis, and in the absence of this information, without undergoing syphilis screening.

## Data Availability

The original contributions presented in the study are included in the article/Supplementary Material, further inquiries can be directed to the corresponding author.
